# Adipose-specific BMP and activin membrane-bound inhibitor (BAMBI) deletion promotes adipogenesis by accelerating ROS production

**DOI:** 10.1074/jbc.RA120.014793

**Published:** 2020-11-23

**Authors:** Xiaochang Chen, Chen Zhao, Yanting Xu, Kuilong Huang, Yulong Wang, Xiaoyu Wang, Xiaoge Zhou, Weijun Pang, Gongshe Yang, Taiyong Yu

**Affiliations:** Key Laboratory of Animal Genetics, Breeding and Reproduction of Shaanxi Province, Laboratory of Animal Fat Deposition & Muscle Development, College of Animal Science and Technology, Northwest A&F University, Yangling, Shaanxi, China

**Keywords:** BAMBI, adipogenesis, ROS production, NADPH oxidase 4, insulin resistance, BAMBI, bone morphogenetic protein and activin membrane-bound inhibitor, BAT, brown adipose tissue, CD, chow diet, DMEM, Dulbecco’s modiﬁed Eagle medium, GM, growth medium, H&E, hematoxylin and eosin, HDL, high-density lipoprotein, HFD, high-fat diet, LDL, low-density lipoprotein, MCE, mitotic clonal expansion, MCP1, monocyte chemoattractant protein 1, NAC, N-acetylcysteine, NADPH, nicotinamide adenine dinucleotide phosphate, NOX, NADPH oxidase, OXPHOS, oxidative phosphorylation, RER, respiratory exchange rate, ROS, reactive oxygen species, RT-qPCR, real-time quantitative PCR, TG, triglycerides, TGF, transforming growth factor, TNFα, tumor necrosis factor α

## Abstract

With the improvement of people's living standards, the number of obese patients has also grown rapidly. It is reported that the level of oxidative stress in obese patients has significantly increased, mainly caused by the increase in reactive oxygen species (ROS) levels in adipose tissue. Studies have shown that the use of siRNA to interfere with bone morphogenetic protein and activin membrane-bound inhibitor (BAMBI) expression could promote adipocyte differentiation, and under hypoxic conditions, BAMBI could act as a regulator of HIF1α to regulate the polarity damage of epithelial cells. In view of these results, we speculated that BAMBI may regulate adipogenesis by regulating the level of ROS. In this study, we generated adipose-specific BAMBI knockout mice (BAMBI AKO) and found that compared with control mice, BAMBI AKO mice showed obesity when fed with high-fat diet, accompanied by insulin resistance, glucose intolerance, hypercholesterolemia, and increased inflammation in adipose tissue. Interestingly, adipose-specific deficiency of BAMBI could cause an increase in the expression level of Nox4, thereby promoting ROS production in cytoplasm and mitochondria and the DNA-binding activity of C/EBPβ and ultimately promoting adipogenesis. Consistently, our findings indicated that BAMBI may be a reactive oxygen regulator to affect adipogenesis, thereby controlling obesity and metabolic syndrome.

In recent years, obesity has prevailed worldwide, and 300 million adults have been classified as obese (BMI >30) (1). Obesity is closely related to many pathological conditions and diseases, including hypertension, type 2 diabetes, dyslipidemia, kidney disease, heart disease, certain types of cancer, acute respiratory distress syndrome, and osteoarthritis (2, 3, 4). Generally, obesity is characterized by excessive accumulation of white adipose tissue, which is produced by the increased size (hypertrophy) or number (hyperplasia) of adipocytes or the combination of hypertrophy and hyperplasia. Therefore, the study of adipocytes differentiation is of great value in alleviating various diseases caused by obesity.

Bone morphogenetic protein and activin membrane-bound inhibitor (BAMBI) is a transmembrane glycoprotein that is highly conserved from human to zebrafish development in vertebrates (5, 6, 7). Because it is highly homologous to the transforming growth factor (TGF) beta superfamily type I receptor, BAMBI is considered to be a pseudoreceptor of the TGFβ-related signaling pathway and acts as a negative regulator of the TGFβ signaling pathway. It has been reported that BAMBI was involved in many aspects of biological processes, such as organ development and disease (8). On the one hand, *in vitro* studies have shown that BAMBI could promote skeletal muscle myogenic differentiation and transformation of myofiber type in C2C12 cells by activating Wnt/β-catenin signaling (9, 10), whereas in myocardium, BAMBI played a critical role in resisting cardiac hypertrophy by inhibiting TGF-β signaling (11). On the other hand, BAMBI also inhibits TGFβ-induced downregulation of estradiol and progesterone, indicating the potential role of BAMBI in reproduction (12). In addition, BAMBI has also been reported to play a key role in the development process of adipose tissue. The downregulated expression of BAMBI by siRNA could inhibit promotion of FGF-1 on lipogenesis (8). Meanwhile, BAMBI was also confirmed to be able to inhibit adipogenesis by a feedback loop, which forms with canonical Wnt/β-catenin signaling (13). However, at present, research on BAMBI is focused on the cellular level, and *in vivo* tests are rarely reported, especially in terms of adipose tissue development.

Reactive oxygen species (ROS) can significantly regulate the process of adipocyte differentiation and then affect the occurrence and development of obesity and related diseases. In cells, ROS is mainly produced under the action of the mitochondrial respiratory system, nicotinamide adenine dinucleotide phosphate (NADPH) oxidases, endoplasmic reticulum stress, certain metabolism, and detoxifying enzymes (14). In the past few years, increasing evidence has supported that ROS is an essential factor for adipocyte differentiation and is considered an important medium for adipogenic hormone-induced C/EBPβ production and adipogenesis (15, 16). Studies have shown that ROS can further promote the DNA binding activity of C/EBPβ by affecting the mitotic clonal expansion (MCE) phase of differentiation and ultimately promote the adipogenic process (17). NADPH oxidase (NOX) is an important enzyme that produces ROS in cells (18). In the NOX family, only NOX4 and p47phox isoforms are involved in adipocyte differentiation (19, 20). In mesenchymal stem cells, drugs that induce differentiation can induce ROS production, and antioxidant treatment and removal of NOX4 can reduce the level of ROS and can prevent the differentiation of adipocytes.

In the present study, we determined whether BAMBI and/or NOX4 activity levels are altered during the development of adipose tissue in mice challenged with high-fat diet (HFD). Further, we utilized a new mouse model in which BAMBI has been deleted specifically in adipocytes to characterize the pathophysiological role of BAMBI in an HFD-induced obesity model. We now show that BAMBI deficiency in adipocyte caused hypertrophy and hyperplasia of adipocytes and an elevated NOX4-derived ROS level during the development of obesity. Moreover, mice with adipocytes deficient in BAMBI show an increased onset of insulin resistance with upregulated adipose tissue inflammation.

Collectively, these data suggest that BAMBI may play a role in adipose tissue development. Such enhanced understanding of the interplay between BAMBI, ROS, and adipocyte differentiation could provide novel directions for the prevention of obesity-associated conditions.

## Results

### Disruption of BAMBI promotes adipose tissue expansion

Despite the fact that the interference efficiency and impacts of BAMBI shRNA lentiviruses had achieved the desired results (data not shown), it was necessary to construct adipocyte-specific BAMBI knockout mice to sufficiently illustrate the function of BAMBI.

**Figure 1:**
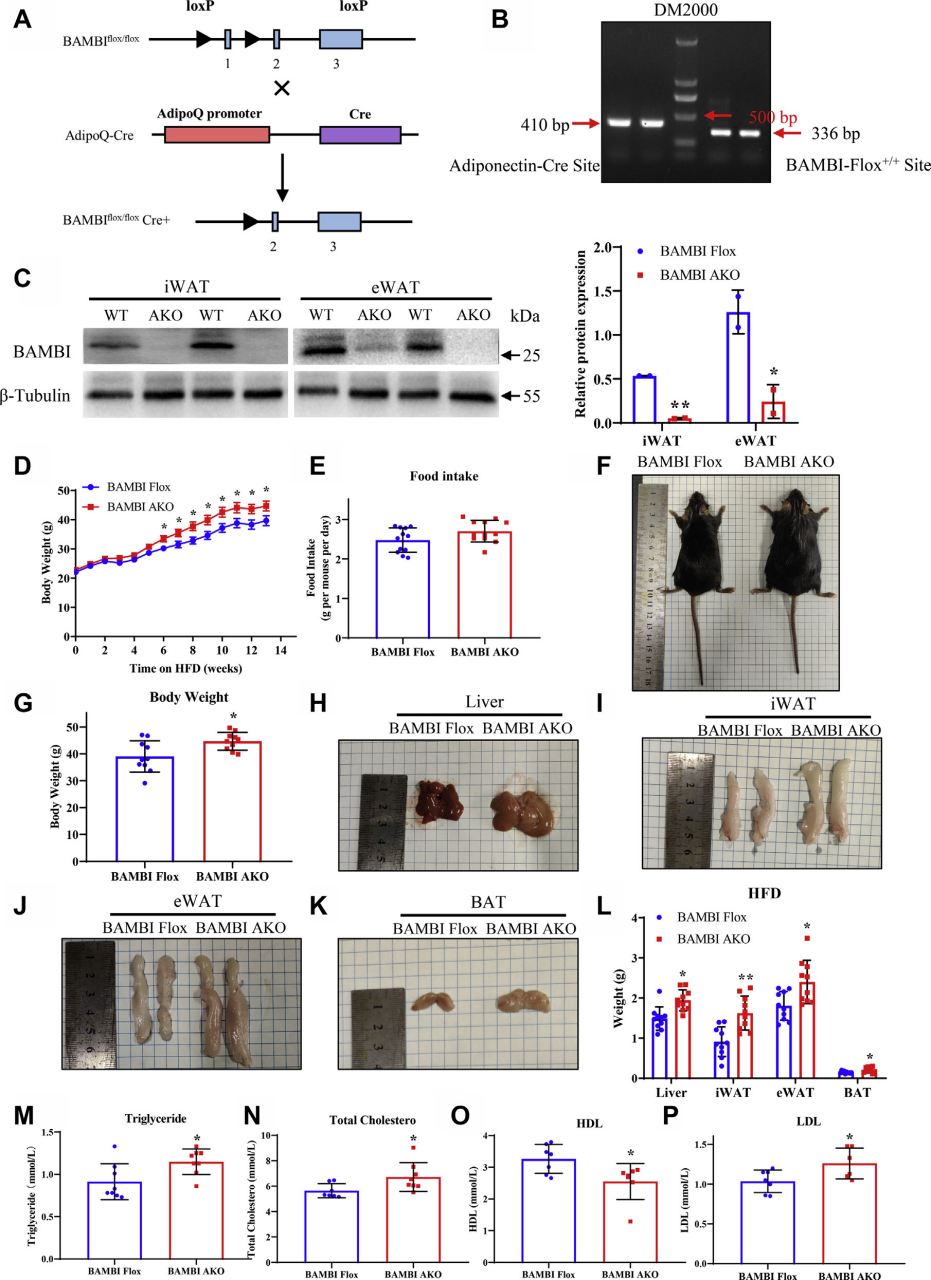
**Generation of adipose-specific BAMBI-knockout mice.***A*, schematic of the Cre-loxP system used to construct the adipose-specific BAMBI AKO mice model. *B*, gel electrophoresis analysis of genotype identification of BAMBI AKO mice. *C*, western blot analysis of BAMBI protein expression in adipose tissue (ingWAT, epiWAT) of 20-week-old BAMBI Flox and BAMBI AKO mice and quantiﬁcation, with β-Tubulin as a control. *D*, body weight and (*E*) food intake statistics of BAMBI Flox and AKO mice fed with HFD diet for 14 weeks (n = 12). *F*, comparison of body shape and (*G*) weight of mice after 14 weeks of HFD feeding (n = 10). *H*–*L*, morphological comparison and weight statistics of the liver, iWAT, eWAT, and BAT after sacrifice of mice (n = 10). *M*–*P*, the serum of mice was collected to detect the statistics of TG, TC, HDL, and LDL (n = 8). Data are represented as the mean ± SD. Signiﬁcance was determined by *t*-test analysis, ∗*p* < 0.05, ∗∗*p* < 0.001.

Therefore, we purchased BAMBI FLOX C57BL/6 mice from The Jackson Laboratory and Adiponectin-Cre C57BL/6 mice from Nanjing Model Animal Research Institute and then generated adipose-specific BAMBI knockout (BAMBI AKO) mice by crossbreeding these two types of mice (Fig. 1*A*). Over the course of a few months, these two mouse lines were continually expanded and crossed, and we ultimately obtained adipose-specific BAMBI knockout mice, called BAMBI AKO mice (Fig. 1*B*). At the beginning, we tested the effect of BAMBI on adipogenesis under chow diet (CD), but unfortunately, we found that there were no significant differences in body weight, liver tissue weight, subcutaneous adipose tissue (iWAT), visceral adipose tissue (eWAT), adipocyte morphology, and adipogenesis marker genes between the two groups of mice (Fig. S1). Therefore, we explored the effect of BAMBI on lipid accumulation with HFD. After 14 weeks of HFD feeding, the knockout efficiencies of BAMBI in iWAT and eWAT were examined. The results showed that BAMBI was knocked out in both iWAT and eWAT (Fig. 1*C*). Upon observing the morphology of the mice, BAMBI AKO mice showed more body weight gain since week 6 under HFD conditions, with no differences in food intake (Fig. 1, *D*–*G*). After the mice were sacrificed and dissected, the effects of BAMBI knockout on tissue morphology were observed. We found that the liver, iWAT, eWAT, and brown adipose tissue (BAT) of the knockout mice were heavier than those of the control mice (Fig. 1, *H*–*L*). In addition, higher serum levels of total cholesterol, triglycerides (TG), and low-density lipoprotein (LDL) and lower levels of high-density lipoprotein (HDL) were detected in BAMBI AKO mice (Fig. 1, *M*–*P*). The above results indicate that the adipose-specific knockout of BAMBI can cause obesity and lipid metabolism disorders under HFD conditions.

### BAMBI ablation results in adipocyte hypertrophy

**Figure 2:**
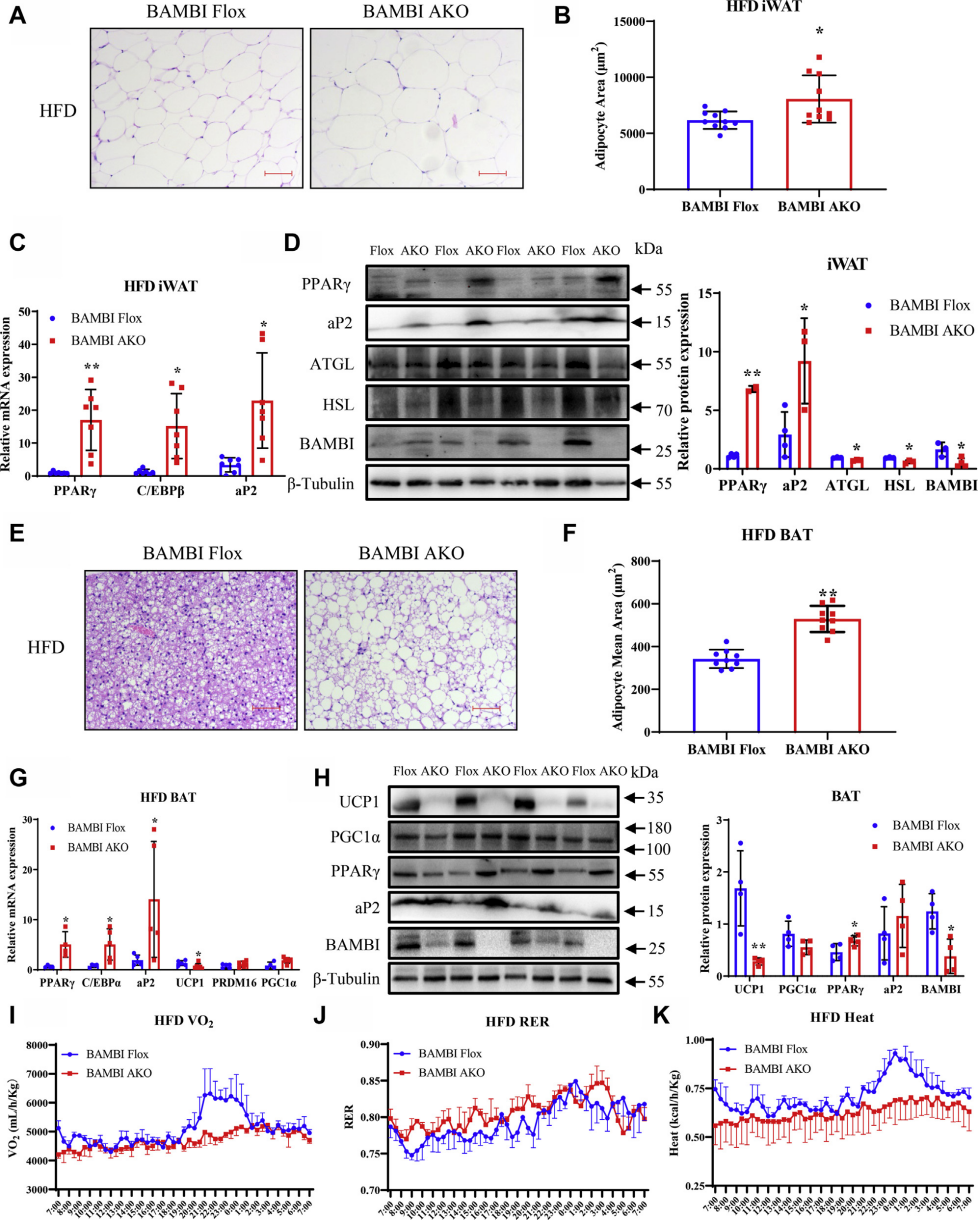
**Adipose-specific BAMBI AKO mice have increased fat deposition and reduced heat production with high-fat diet.***A* representative image of H&E-stained sections of iWAT tissues from BAMBI Flox and BAMBI AKO mice with HFD diet, scale bar: 50 μm. *B*, adipocyte area statistics of iWAT from HFD-fed BAMBI Flox and AKO mice (n = 10). *C*, comparison of mRNA expression levels of Pparγ, C/ebpβ, Ap2 in iWAT of BAMBI-Flox and AKO mice, β-actin as a correction (n = 7), scale bar: 50 μm. *D*, western blot analysis of PPARγ, AP2, ATGL, HSL, and BAMBI expression in iWAT of BAMBI Flox and AKO mice and quantiﬁcation, with β-Tubulin as a control (n = 4). *E*, representative image of H&E-stained sections of BAT tissues from BAMBI Flox and AKO mice with HFD diet. Scale bar: 50 μm. *F*, adipocyte area statistics of BAT from HFD-fed BAMBI Flox and AKO mice (n = 9). *G*, comparison of mRNA expression levels of Pparγ, C/ebpβ, Ap2, Ucp1, Prdm16, and Pgc1α in BAT of BAMBI Flox and AKO mice, with β-actin as a control (n = 6). *H*, western blot analysis of UCP1, PGC1α, PPARγ, aP2, and BAMBI expression in BAT of BAMBI Flox and AKO mice and quantiﬁcation, with β-Tubulin as a control (n = 4). *I*–*K*, metabolism studies of control and BAMBI AKO mice fed an HFD: oxygen consumption (*I*), respiratory exchange ratio (RER), (*J*) and heat production (*K*) (n = 4). Data are represented as the mean ± S.D. Signiﬁcance was determined by *t*-test analysis, ∗*p* < 0.05, ∗∗*p* < 0.001.

In general, the increased size of adipocytes (hypertrophy) causes an increase in adipose tissue mass. To reveal the mechanism of increased adiposity in BAMBI AKO mice, adipocyte size was measured in the adipose tissue of HFD-fed control and BAMBI AKO mice. Hematoxylin and eosin (H&E) staining indicated that adipocytes were larger in both iWAT and BAT of BAMBI AKO compared with control mice (Fig. 2, *A* and *E*). Further adipocyte area quantification supported the result of increased adipocyte size in BAMBI AKO adipose tissue (Fig. 2, *B* and *F*). Furthermore, we detected the expression levels of adipogenic and thermogenic marker genes in iWAT or BAT of BAMBI AKO mice at the mRNA and protein levels, and the results were consistent with those obtained by H&E staining, thereby suggesting that increased fat mass in BAMBI AKO mice was caused by adipocyte hypertrophy (Fig. 2, C, D, G and H). We also evaluated the effect of BAMBI ablation in adipose tissue on basic metabolic activity. Under HFD condition, BAMBI AKO mice showed significantly reduced oxygen consumption and heat production compared with controls, although respiratory exchange rate (RER) had no significant change (Fig. 2, I–*K*), indicating that the decreased resting metabolic rate may be the main cause of obesity in BAMBI AKO mice.

### Adipose-specific BAMBI deletion exacerbates insulin resistance

**Figure 3:**
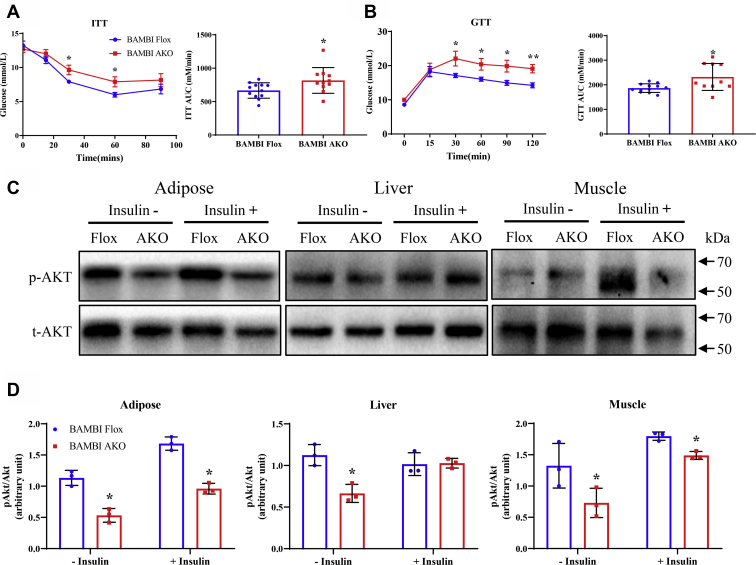
**Adipose-speciﬁc BAMBI deletion exacerbates insulin resistance.***A*–*B*, insulin tolerance test (*A*) and glucose tolerance test (*B*) in control and BAMBI AKO mice fed an HFD, Right panel, area under the curve (n = 12). *C*–*D*, western blot analysis of Akt phosphorylation levels and and in the adipose tissue, liver, and muscle of control and BAMBI AKO mice with HFD (n = 6). Data are represented as the mean ± SD. Signiﬁcance was determined by Student’s *t*-test analysis, ∗*p* < 0.05.

Glucose intolerance and insulin resistance are closely related to obesity (21, 22, 23). In the present study, decreased adipose BAMBI expression caused impaired glucose tolerance and insulin resistance in HFD-fed mice, but not in CD-fed mice (data not shown). Then, we assessed the effect of adipose-specific BAMBI deletion on glucose homeostasis and insulin sensitivity in the HFD-fed group. Fortunately, BAMBI AKO mice exhibited glucose intolerance and insulin resistance compared with BAMBI-Flox mice (Fig. 3, *A*–*B*). Changes in the AKT signaling pathway are often used to measure whether the insulin signaling pathway is impaired. To investigate the damage to the insulin signaling pathway after BAMBI knockout, we examined the expression levels of phosphorylated insulin-stimulated Akt Ser473 in iWAT, liver, and muscle of mice. Insulin-induced Akt phosphorylation was attenuated in the adipose tissue of BAMBI AKO mice, suggesting that BAMBI knockout could downregulate the HFD-induced insulin sensitivity. (Fig. 3, *C*–*D*). These results indicate that BAMBI knockout could exacerbate HFD-induced insulin resistance in adipose tissue.

### BAMBI knockout accelerates preadipocyte differentiation of iWAT and BAT

**Figure 4:**
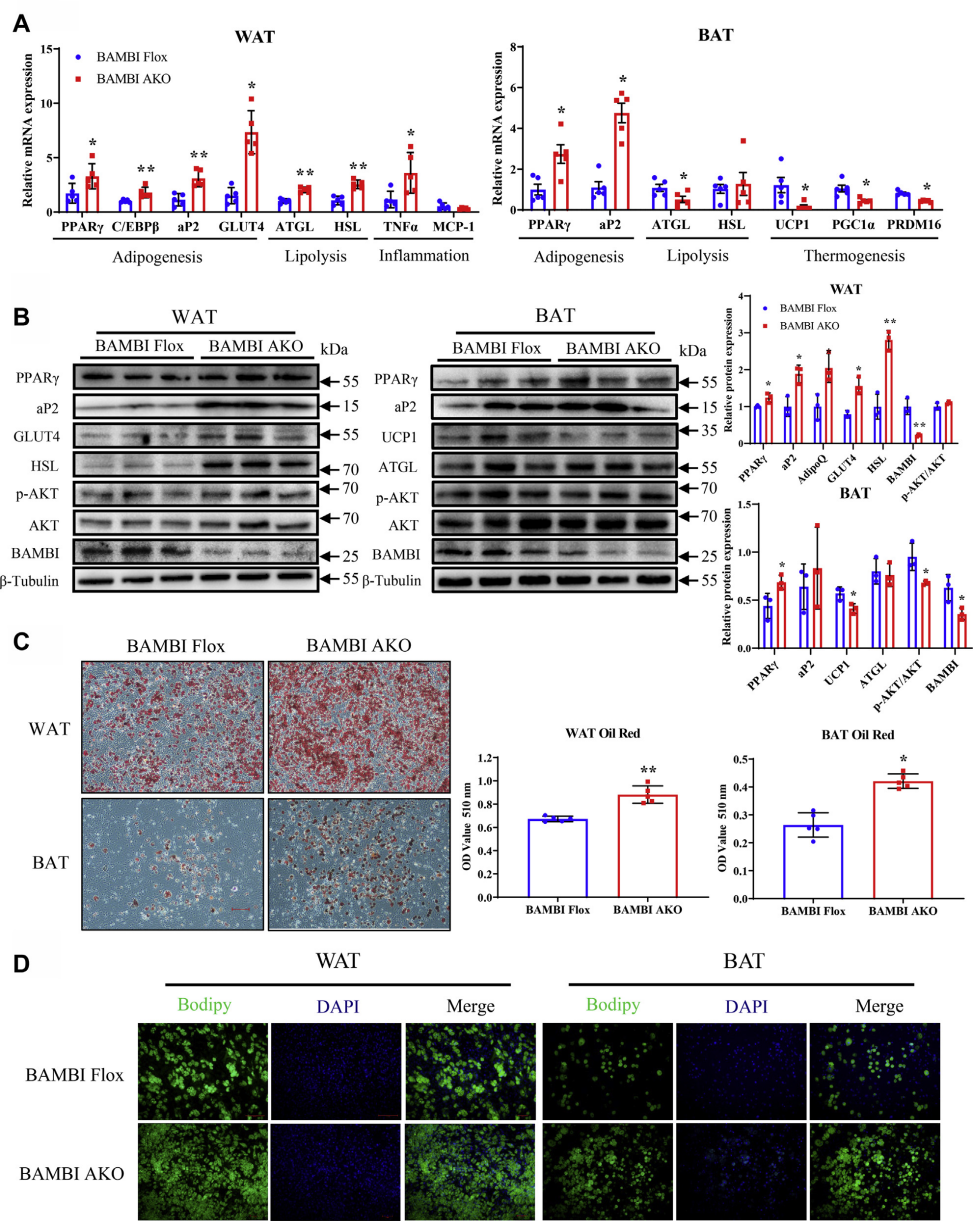
**Knockout of BAMBI enhances the adipogenesis of adipocytes.***A*, the RT-qPCR results of adipogenesis-, lipolysis-, inflammation-, and thermogenesis-related genes of in adipocyte isolated from iWAT and BAT, with β-actin as a control (n = 5). *B*, western blot analysis of PPARγ, aP2, GLUT4, HSL, p-ATK, AKT, and BAMBI expression in adipocyte isolated from iWAT and BAT of BAMBI Flox and AKO mice and quantiﬁcation, with β-Tubulin as a control (n = 3). *C*, quantitative results of Oil Red O staining and quantiﬁcation in iWAT and BAT adipocytes of BAMBI Flox and AKO mice (n = 5), scale bar: 200 μm. *D*, bodipy staining in iWAT and BAT adipocyte of BAMBI-Flox and AKO mice, DAPI: nucleus, Scale bar: 200 μm. Data are represented as the mean ± SD. Signiﬁcance was determined by *t*-test analysis, ∗*p* < 0.05, ∗∗*p* < 0.001. RT-qPCR, real-time quantitative PCR.

Next, to further verify the results obtained *in vivo* and to construct a cell model for mechanistic studies, we isolated preadipocytes from iWAT and BAT of control and BAMBI AKO mice. Consistent with *in vivo* experiments, the preadipocytes isolated from iWAT and BAT of KO mice showed higher differentiation potential. In addition, we noticed significant increases in lipogenesis genes (*e.g.*, PPARγ, aP2, C/EBPβ), lipolysis genes (*e.g.*, ATGL, HSL), and inflammation genes (*e.g.*, TNFα) in BAMBI knockout preadipocytes (Fig. 4, *A*–*B*). The results confirmed the participation of adipocyte differentiation in WAT expansion in the adipose of BAMBI-depleted mice. At the same time, preadipocytes separated from BAT of BAMBI-deficient mice were more easily differentiated into adipocytes with higher expression levels of lipogenesis genes (*e.g.*, PPARγ, aP2) and lower expression levels of thermogenesis genes (*e.g.*, UCP1, PGC1α, PRDM16) (Fig. 4, *A*–*B*). The results of Oil Red O and Bodipy staining also verified the above results at the mRNA and protein levels, suggesting that this cell model could be used for subsequent studies (Fig. 4, *C*–*D*).

### BAMBI deficiency in adipose tissue potentiates ROS-related pathways

**Figure 5:**
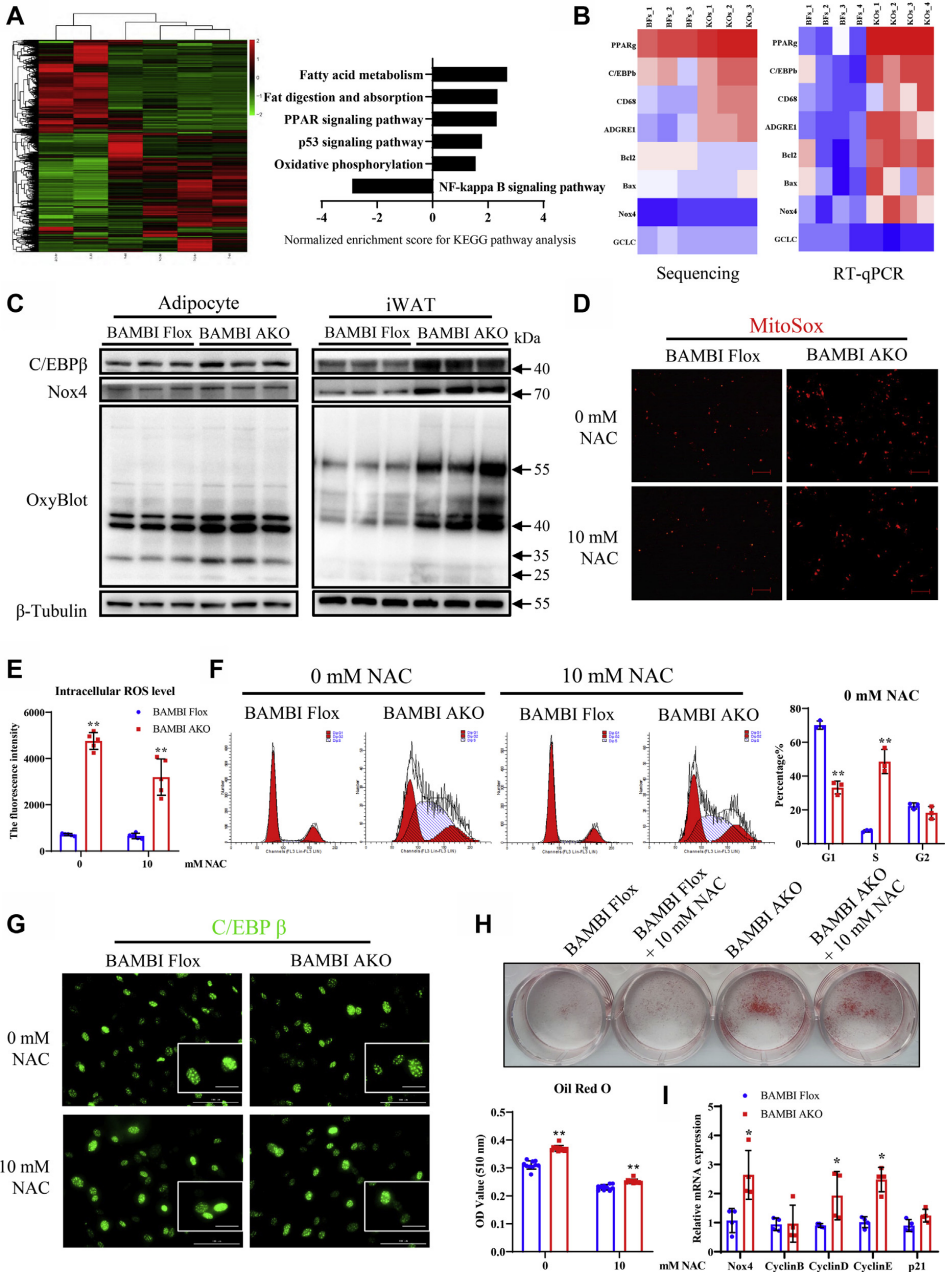
**Adipose-specific BAMBI deletion promotes adipogenesis by accelerating ROS production.***A*, heatmap representing global gene expression in iWAT, eWAT, and BAT of BAMBI Flox and BAMBI AKO mice after 12 weeks of HFD (n = 3). Gene enrichment analyses of the significantly changed pathways were shown as normalized enrichment score (NES) plots. *B*, heatmap representing the ‘‘remodeling’’ genes upregulated in iWAT of the BAMBI AKO mice after 12 weeks of HFD (n = 3). RT-qPCR analysis of Pparγ, C/ebpβ, CD68, Adgre1, Bcl2, Bax, Nox4, and Gclc in iWAT from BAMBI Flox and BAMBI AKO mice after 12 weeks of HFD (12 weeks HFD, n = 4). *C*, western blot analysis of C/EBPβ, NOX4, and OxyBlot in adipocyte and iWAT of control and BAMBI AKO mice with HFD. Protein levels were normalized to β-Tubulin level (n = 3). *D*, immunofluorescence staining of MitoSox in iWAT adipocytes of the BAMBI AKO compared with that of Flox mice after 24 h of adipogenic induction in the absence (up panel) or presence of N-acetyl cysteine (NAC, 10 Mm, down panel), scale bar: 200 μm. *E*, the fluorescence intensity of intracellular ROS level in iWAT adipocytes of the BAMBI AKO compared with Flox mice after 24 h of adipogenic induction in the presence of 0 or 10 mM NAC (n = 5). *F*, changes in DNA content were analyzed by FACS after 24 h of induction in the presence of 0 or 10 mM NAC (n = 3). *G*, immunofluorescence staining of C/EBPβ in preadipocytes after adipogenic induction for 24 h in the absence (up panel) or presence of NAC (10 Mm, down panel), scale bar: 100 μm, scale bar in magnified insets: 50 μm. *H*, representative image of Oil Red O-stained of iWAT adipocytes from BAMBI Flox and BAMBI AKO mice after adipogenic induction in the absence (up panel) or presence of N-acetyl cysteine (10 Mm, down panel) at 8 days (n = 6). *I*, RT-qPCR analysis of mRNA levels of proliferation-related genes in adipocytes isolated from iWAT in control and BAMBI AKO mice (n = 5). Data are represented as the mean ± SD. Signiﬁcance was determined by Student’s *t*-test analysis, ∗*p* < 0.05, ∗∗*p* < 0.001. RT-qPCR, real-time quantitative PCR.

RNA sequencing revealed that 1356 transcripts were significantly changed in iWAT from BAMBI AKO mice relative to BAMBI Flox mice (Fig. 5*A*). Among these changed transcripts were genes involved in adipogenesis, apoptosis, inflammation, and redox modulation, including the PPAR signaling pathway, the NF-kappa B signaling pathway, and oxidative phosphorylation, suggesting a crucial function of BAMBI in adipocyte remodeling (Fig. 5*A*). Interestingly, our transcriptome analysis revealed that iWAT of BAMBI AKO mice is characterized by significant changes in genes involved in redox modulation, such as Nox4 and GCLC (Fig. 5*B*). The significant changes of Nox4 and p53 in isolated adipocytes and iWAT of BAMBI AKO mice were confirmed by real-time quantitative PCR (RT-qPCR) analyses (Fig. 5*B*). Consistent with these observations, the protein levels of C/EBPβ, NOX4, mitochondrial oxidative phosphorylation (OXPHOS) were markedly increased in adipocytes and iWAT of BAMBI AKO mice compared with BAMBI Flox mice, suggesting that BAMBI may regulate the adipogenesis process by increasing the intracellular ROS (Fig. 5*C*). To confirm this hypothesis, we measured the mitochondrial and intracellular ROS levels in iWAT adipocytes after 24 h of induction (Fig. 5*D*, up panel, and Fig. 5*E*). In isolated adipocytes, the mitochondrial and intracellular ROS level were significantly increased in BAMBI-deficient adipocyte. All of the above results suggested that knockout of BAMBI in adipocytes could upregulate the mitochondrial and intracellular ROS level after 24 h of induction.

### BAMBI knockout facilitates mitotic clonal expansion and promotes the activation of C/EBPβ

Next, we examined how cell cycle events during MCE are affected by ROS production. Upon induction, growth-arrested iWAT preadipocytes synchronously reenter the cell cycle, resulting in the detection of dividing cells (G2/M) by FACS analysis after 24 h of induction (Fig. 5*F*). Interestingly, BAMBI knock in preadipocytes caused an increase in the S population during adipocyte differentiation (Fig. 5*F*).

Previous investigations have shown that activation of C/EBPβ was associated with ROS production, and it was only bound to its target sites on DNA until cells entered the S phase at centromeres, resulting in a characteristic “punctate” pattern in immunofluorescence studies. As shown in Figure 5*G*, the punctate pattern of C/EBPβ was detected at the 24 h time point in BAMBI AKO preadipocytes, while C/EBPβ still remained diffuse in the control group. Conversely, the general antioxidant NAC could cause the opposite phenomenon (Fig. 5*G*). The results of Oil Red O staining showed that the use of NAC still inhibited the normal differentiation of preadipocytes. These results indicated that BAMBI knockout facilitated MCE and promoted the activation of C/EBPβ. Moreover, advancing cell cycle progression was confirmed by assaying the expression levels of Cyclin D, Cyclin E, p21, and p27 as shown in Figure 5*I*, suggesting that BAMBI is important for the progression of MCE during adipocyte differentiation.

### N-acetylcysteine inhibits adipogenic differentiation caused by BAMBI knockout

To further verify our previous hypothesis, we used the antioxidant N-acetylcysteine (NAC) to suppress ROS production in preadipocytes at the concentration of 10 mM. The treatment method involved adding the NAC working solution in proportion to the cocktail method inducing solution I and then adding the inducing solution I to the cell culture plate for processing. From the immunofluorescence staining of MitoSox, after adding NAC, the red fluorescent bright spot of MitoSox was significantly reduced, indicating that NAC inhibited the ROS produced by mitochondria (Fig. 5*D*, down panel). Based on the ROS measurement results, after adding NAC, the fluorescence intensity of the DCF probe in the precursor adipocyte cells was significantly reduced, indicating that NAC inhibited the ROS produced in the cells (Fig. 5*E*). As shown in Figure 5*F*, the results of flow cytometry showed that after adding NAC, the proportion of S phase in BAMBI AKO preadipocytes decreased significantly, indicating that NAC inhibited the MCE phase of preadipocytes. After adding NAC, the immunofluorescence characteristics of C/EBPβ showed a “cloudy” distribution compared with when NAC was not added, indicating that the addition of NAC could delay the DNA-binding activation of C/EBPβ with downstream target genes (Fig. 5*G*, down panel). The results of Oil Red O staining also showed that the Oil Red O staining area was significantly reduced, and the OD value of extracted Oil Red O at 510 nm was also decreased significantly after adding NAC, indicating that NAC inhibited the adipogenic differentiation of preadipocytes (Fig. 5*H*).

### HFD-fed BAMBI AKO mice show increased hepatic steatosis

**Figure 6:**
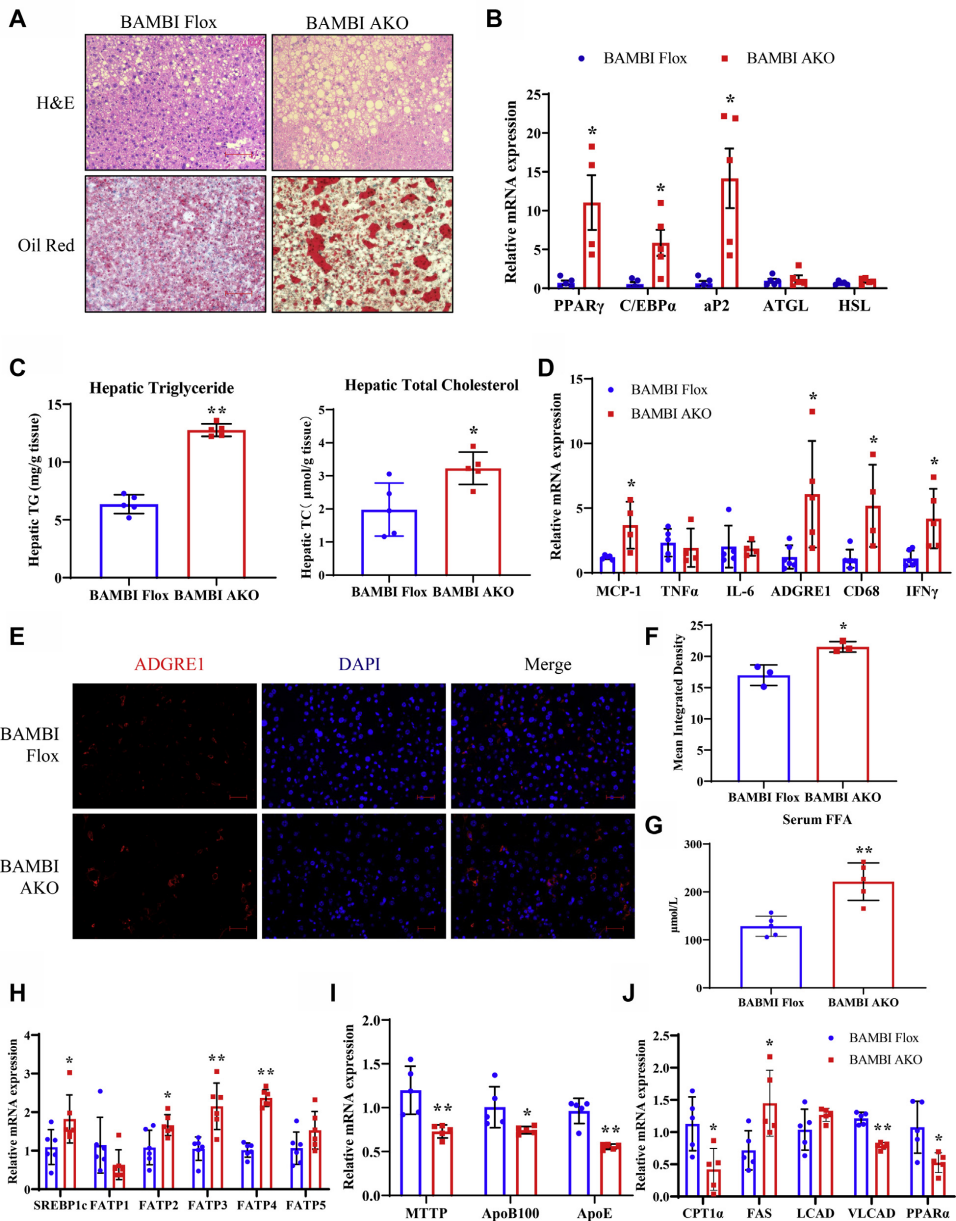
**HFD-fed BAMBI AKO mice show increased hepatosis.***A*, representative image of H&E- and Oil Red O-stained sections of liver tissues from BAMBI Flox and BAMBI AKO mice with HFD diet. Scale bar: 50 μm. *B*, RT-qPCR analysis of mRNA levels of lipogenesis-related genes in livers from control and BAMBI AKO mice fed an HFD (n = 5). *C*, levels of triglycerides and total cholesterol in the livers of control and BAMBI AKO mice fed an HFD (n = 5). *D*, RT-qPCR analysis of mRNA levels of inﬂammation-related genes in the livers from control and BAMBI AKO mice fed an HFD (n = 5). *E*–*F*, immunofluorescence staining and mean integrated density of ADGRE1 in the liver. DAPI: nucleus. Scale bar: 200 μm. *G*, the serum FFA level of control and BAMBI AKO mice (n = 5). *H*–*J*, RT-qPCR analysis of mRNA levels of lipid synthesis-, VLDL packaging function-, and fatty acid β-oxidation-related genes in the livers from control and BAMBI AKO mice fed an HFD (n = 5) Data are represented as the mean ± SD. Signiﬁcance was determined by Student’s *t*-test analysis, ∗*p* < 0.05, ∗∗*p* < 0.001. RT-qPCR, real-time quantitative PCR.

Given that hepatic steatosis is closely associated with obesity and insulin resistance, we assessed the impact of adipose BAMBI deletion on hepatic lipid deposition. HFD-fed mice showed more lipid accumulation in the liver than CD-fed mice and less lipid accumulation than BAMBI AKO mice under an HFD diet (Fig. 6*A*). The mRNA levels of Pparγ, C/ebpα, Ap2 were significantly increased in the livers of BAMBI AKO mice, but Atgl and Hsl expression levels were changed only slightly (Fig. 6*B*). Furthermore, liver TG and total cholesterol levels were significantly higher in BAMBI AKO mice relative to controls (Fig. 6*C*). Altogether, these data suggested that loss of BAMBI in adipocyte exacerbated HFD-induced liver steatosis in part due to increased fatty acid uptake and lipogenesis.

In addition, the mRNA levels of monocyte chemoattractant protein 1 (Mcp1), tumor necrosis factor α (Tnfα), adhesion G-protein-coupled receptor E1 (Adgre1), CD68, and interferon γ (Ifnγ) were upregulated in BAMBI AKO liver (Fig. 6*D*). Furthermore, immunofluorescent staining of macrophage marker ADGRE1 revealed increased number of macrophages in the livers of HFD-fed BAMBI AKO mice (Fig. 6, *E*–*F*). Thus, adipose-specific BAMBI deficiency may cause increased inflammation in liver.

Nonalcoholic fatty liver refers to the excessive accumulation of fat (mainly triacylglycerol) in liver cells due to various reasons. There are many causes of nonalcoholic fatty liver, mainly related to metabolic syndrome (obesity, diabetes, high blood pressure, hyperlipidemia, etc.). In our study, the concentration of serum-free fatty acid and the mRNA expression of cholesterol-binding regulatory binding protein (Srebp1c), fatty acid transporter (Fatp2, Fatp3, Fatp4), and fatty acid synthesis–related genes such as fatty acid synthase (Fas) in the BAMBI knockout group mice liver tissues were significantly higher than those of control group, while the expression of Atgl has no difference, suggesting that the increased fatty acid uptake and lipid synthesis in liver are important reasons for the aggravation of liver tissue steatosis in BAMBI AKO mice (Fig. 6*G*, *H*). On the other hand, the mRNA expression level of Mttp, ApoB100, and ApoE in AKO mice liver was significantly decreased than that of control group, suggested that the disorder of VLDL packaging function may cause the increase of TG content in liver tissue of BAMBI AKO mice (Fig. 6*I*). From the perspective of fatty acid oxidation, RT-qPCR results showed that the mRNA expression of Cpt1 and Pparα in BAMBI knockout mice liver was significantly lower than that of control mice, indicated that BAMBI AKO mice liver fatty acid oxidation ability is impaired (Fig. 6*J*). Based on all the above results, we believe that deficiency of BAMBI gene may indirectly cause hepatic steatosis by promoting the release of more FA from hypertrophic adipose tissue, rather than directly regulating the ability of the adipogenesis in liver.

## Discussion

In the current study, we established BAMBI adipose-specific knockout mice through the Loxp-Cre system. Under HFD induction, this model showed highly consistent phenotypes with individuals with obesity. BAMBI AKO mice exhibited white adipose expansion with *de novo* lipogenesis and detrimental inflammation, accompanied by increased ectopic lipid deposition and decreased insulin sensitivity. Consistently, the same situation was found in BAT.

Mechanistically, we demonstrated that BAMBI could mediate the adipogenesis process by increased ROS level. Adipose-specific knockout of BAMBI could cause an increase in the expression level of Nox4, thereby promoting the ROS production in the cytoplasm and mitochondria and the DNA-binding activity of C/EBPβ, ultimately promoting adipogenesis. In other words, our findings indicated that BAMBI could function as a reactive oxygen regulator to affect adipogenesis.

In our recent study, BAMBI protein is highly expressed in skeletal muscle and is detected only in the cytosolic fraction of resting muscle. In addition, BAMBI proteins are colocalized in fast-twitch (glycolytic) fibers, rather than in slow-twitch (oxidative) fibers. Compared with cytoplasmic capture in resting muscle, BAMBI protein is enriched on the cell membrane during muscle growth and regeneration, suggesting that important signal pathways mediated by BAMBI may be an important component of muscle growth and regeneration (10). It can be seen from the above results that BAMBI plays an important regulatory role in many physiological and pathological processes of the body, including lipid accumulation, but at present, the effect of BAMBI on adipogenesis has not been clearly clarified *in vivo*. Recent research results show that BAMBI can promote the polarization of M2 macrophages to M1 macrophages through the TGF-β/Smad signaling pathway, and under hypoxic conditions, BAMBI could function as a regulator of HIF1α to promote TGFβ signaling and then induced polar disruption of epithelial cells, indicating that BAMBI may affect adipogenesis through inflammation or reactive-oxygen-related pathways (24, 25). Thus, here we generated fat-specific BAMBI knockout mice and found that BAMBI knockout can lead to HFD-induced obesity and insulin resistance, thereby deepening our understanding of BAMBI function.

Although there is still some controversy in the literature about the role of ROS on adipogenesis, for the time being, ROS is still considered to promote adipogenesis (26). First, what we should know is that as early as 20 years ago, there was evidence that ROS generation was observed during the process of adipogenesis (27), and increasing amounts of results now consider ROS as a necessary factor for adipocyte differentiation (16). Researchers have found that ROS levels gradually increase after adipogenic induction, and treatment with antioxidants or interference of Nox4 could inhibit adipocyte differentiation in mesenchymal stem cells (28). Moreover, ROS can also be indirectly involved in adipocyte differentiation. For instance, Younce *et al.* (29) showed that upregulation of MCP-1 inducible protein (MCPIP) could increase ROS/RNS levels and iNOS expression, while inhibition of ROS production attenuated MCPIP-induced adipogenesis in 3T3-L1 preadipocytes. In addition, another more important point is that C/EBPβ and PPARγ are the main factors in the ROS-mediated adipogenesis process. For example, when cocktail induction is used to induce preadipocytes, ROS can activate C/EBPβ and form dimers by forming intermolecular disulfide bonds, thereby enhancing its DNA-binding activity (30). Moreover, when 3T3-L1 cells were treated with H_2_O_2_, differentiation would accelerate with increasing PPARγ expression (17). In this study, we have demonstrated that BAMBI adipose-specific knockout can promote the production of ROS in the cytoplasm and mitochondria, thereby promoting C/EBPβ to perform its function and ultimately promoting adipogenesis, which may reveal the potential regulation mechanisms of BAMBI during adipogenesis.

ROS are produced during the adipogenesis process, and the production of ROS is necessary for adipogenesis. Therefore, inhibiting the production of ROS may inhibit adipogenesis, thereby suppressing the expansion of adipose tissue. Studies have reported that ROS is mainly produced by the mitochondrial respiratory system, NADPH oxidase (Noxs), and other systems in cells. On the one hand, after the preadipocytes are induced to enter the differentiation phase, the division, replication, and metabolism of mitochondrial would suddenly and sharply increase. As the adipocytes continue to differentiate, this change would gradually stabilize and eventually return to normal levels. As an essential signal or by-product, both increased mitochondrial activity and enhanced OXPHOS could lead to more ROS production. On the other hand, NADPH oxidase (NOX) is an important enzyme that produces ROS in cells (31), of which there are seven subtypes in mammals. In the NOX family, only the NOX4 and p47phox subtypes are involved in adipocyte differentiation. It has been demonstrated that the increase in ROS is often accompanied by an increase in NOX4 activity in hypertrophic adipose tissue (32), and the increased NOX4 activity was closely related to the increase in its mRNA expression (33), which is consistent with our findings. Therefore, the increased expression of NOX4 due to BAMBI knockout may promote the obese phenotype in BAMBI AKO mice. However, at this time, we do not know if there is any interaction between BAMBI and NOX4 and how they function. Thus, the further study will focus on this question. Increasing evidence has shown that impaired lipolysis of adipocytes could lead to the infiltration of proinflammatory immune cells and the development and deterioration of insulin resistance in the metabolic tissues of obese individuals. In our study, BAMBI deficiency worsens insulin resistance and inflammatory infiltration in adipose tissue. At the same time, obese BAMBI AKO mice also exhibited severe hepatic steatosis, which is characterized by excessive accumulation of TG.

In summary, we demonstrate that BAMBI knockout will produce more ROS in adipocytes, resulting in adipose tissue hypertrophy. Adipose-specific deletion of BAMBI promoted HFD-induced obesity, impaired adipose function, and deteriorated glucose intolerance and insulin resistance. Thus, BAMBI may be a potential therapeutic target for ameliorating obesity and obesity-related metabolic disorders.

## Experimental procedures

### Animals

BAMBI^flox/flox^ (#009389) mice were purchased from Jackson laboratories (Bar Harbor, ME), and AdipoQ-Cre mice (#024671) were purchased from the model animal resource information platform (34). BAMBI-AKO mice were generated by crossbreeding BAMBI^flox/flox^ mice with AdipoQ-Cre mice. BAMBI^flox/flox^:AdipoQ-Cre^−^ mice were used as controls. Starting at 8 weeks of age, mice were fed an HFD (TP23400; Research Diets) consisting of 60% fat or normal CD (D12450B; Research Diets), and body weight was monitored weekly. Mice were housed on a 12-h light/dark cycle and given *ad libitum* access to food and water. All animal protocols were approved by the EAMC (Committee of Experimental Animal Management) at Northwest Agriculture and Forestry University, China.

### Preadipocyte differentiation

Preadipocytes were isolated from iWAT or BAT of 4-week-old mice as described previously (35). In briefly, the fat pad was cut into approximately 1 mm^3^ pieces, and then, the chopped fat tissue was digested for 40 to 50 min with 0.15% collagenase I (270 U/mg; Gibco, Carlsbad, CA) and Dulbecco’s modiﬁed Eagle medium (DMEM; Gibco, Carlsbad, CA) in a water bath oscillator at 37 °C. After terminating digestion, the digested samples were filtered through 70 and 200 mesh filters. The cells were then washed three times, subjected to 1500 rpm centrifugation for 5 min, and were cultured in growth medium (GM, DMEM/F12 with 10% FBS) at 37 °C in a humidified atmosphere with 5% CO_2_. After 24 h, the cells were washed twice using PBS and the GM was replaced. After that, the culture medium was changed every 2 days. When the white preadipocytes reached 100% confluence, they were cultured with DMEM/F12, supplemented with 5 μg/ml insulin, 1 μM dexamethasone, and 0.5 mM isobutyl methylxanthine (IBMX, Sigma-Aldrich, St Louis, MO) for 2 days. Then the medium was changed into DMEM/F12 with 10% FBS and 5 μg/ml insulin for another 6 days to maintain differentiation. The brown preadipocytes were cultured in brown adipocyte differentiation medium I (DMEM/F12 with 10% FBS and added 0.5 mmol/l IBMX, 1 μmol/l Dex, 5 μg/ml insulin, 1 nmol/l T3, 125 nmol/l Indol, and 1 μmol/l rosiglitazone). The cells were switched to brown adipocyte differentiation medium II (DMEM/F12 with 10% FBS and 5 μg/ml insulin and 1 nmol/l T3) after 2 days. Then, the culture medium was changed every other day and collected at different time points.

### White adipose tissue and liver histology analysis

Adipose tissue and livers were isolated and fixed in 4% paraformaldehyde and maintained at 4 °C until use. The fixed tissues were dehydrated and processed for paraffin embedding, and 5-μm sections were stained with H&E. Adipocyte size was determined by using ImageJ (US National Institutes of Health), measuring a minimum of 300 cells per group. Immunofluorescence and immunohistochemical staining were performed with ADGRE1 antibody (Abcam, ab16911, 1:50). Livers were isolated and frozen in OCT embedding medium; 10-μm sections were used for Oil Red O staining.

### Lipid profile assays

Mice were fasted overnight, and angular vein blood was collected. Serum samples were immediately sent to the Yangling Demonstration Zone Hospital for concentration detection of TG, cholesterol, HDL, and LDL. The TG concentration of the liver was measured by a TG content detection kit (Solarbio Life Science, BC0625, Beijing, China). And the total cholesterol content detection kit (Solarbio Life Science, BC1980, Beijing, China) was used to measure total cholesterol content of the liver.

#### Metabolic studies

For the glucose tolerance test, mice were fasted overnight and then received an intraperitoneal (i.p.) injection of glucose (0.75 g/kg body weight). For the insulin tolerance test, mice were fasted for 4 h before receiving an i.p. injection of insulin (1.5 U/kg body weight). Blood glucose concentrations were measured at 0, 15, 30, 60, 90, and 120 min after glucose or insulin injection. Acute insulin challenge experiments were performed on anesthetized mice fasted for 4 h. At 20 min after an i.p. injection with insulin (2 U/kg body weight), the remaining liver, muscle, and adipose tissue were snap-frozen for subsequent protein extraction.

### Real-time quantitative PCR (RT-qPCR)

Total RNA was extracted from WAT or liver using TRIzol reagent (Invitrogen, Carlsbad, CA). Total RNA was extracted from differentiated stromal vascular fractions by using the RNeasy Lipid Tissue Mini kit (Qiagen, Valencia, CA, USA). An amount of 1 μg RNA was reverse-transcribed into cDNA by using the PrimeScript RT Reagent Kit (Takara Biomedical Technology). PCR amplification was performed using the SYBR PCR mix (Takara Biomedical Technology Co). The primers for quantitative PCR are listed in Table S1.

### Western blot analysis

Briefly, 15 μg samples of total lysates from tissues or cells were run on a 10% SDS-PAGE gel and immunoblotted with the primary antibodies (1:1000) to BAMBI (ThermoFisher, PA5-38027), β-Tubulin (Sungene Biotech, KM9003), PPARγ (Abcam, ab3442), aP2 (Santa, Sc-271529), ATGL (CST, 2138), HSL (CST, 4107), Akt (CST, 4691), p-Akt (ser473,CST, 4060), UCP1 (Abcam, ab10983), PGC1α (CST, 2178), C/EBPβ (Santa, sc-7962), oxidative phosphorylation (OXPHOS) (Abcam, ab110413), and Nox4 (ABclonal, A11274). The intensity of bands was measured using ImageJ. All experiments were repeated at least three times and mean values were derived. All the uncropped blots are included in the Source Data file.

### RNA-seq analysis

Total RNA was extracted from control and BAMBI-deficient adipose tissues. The transcriptome sequencing experiments were performed by Novogene Company (Beijing, China). The transcriptome library for sequencing was generated using a KAPA-Stranded RNA-Seq Library Prep Kit (Illumina) following the manufacturer’s recommendations. The clustering of the index-coded samples used the KAPA RNA Adapters set1/set2 for Illumina. After clustering, the libraries were sequenced on Illumina HiSeq X Ten platform using a (2 × 150 bp) paired-end module. The differentially expressed genes were identified with *p* value <0.05 and a fold-change of >1.5 between two groups.

### Measurement of ROS level

Mitochondrial ROS were determined using MitoSox Red mitochondrial superoxide indicator (ThermoFisher, M36008). After inducing differentiation for 24 h, the medium was removed, and 5 μM MitoSox reagent working solution was then loaded. After 10 min of incubation with MitoSox reagent working solution in the dark at 37 °C, the cells were washed gently three times with warm buffer. The stained cells were imaged using a NIKON fluorescence microscope (Nikon Eclipse, Tokyo, Japan).

Intracellular ROS were determined using a Reactive Oxygen Species Assay Kit (Solarbio Life Science, CA1410, Beijing, China). The method was performed according to the manufacturer's manual. In briefly, DCFH-DA was diluted 1:1000 with serum-free medium. The medium was removed, an appropriate volume of 10 μmol/l DCFH-DA was added, and cells were incubated at 37 °C for 20 min. Next, the cells were washed three times with serum-free cell culture medium to sufficiently remove DCFH-DA. Finally, the fluorescence intensity was analyzed using a Synergy H1 Hybrid Multi-Mode Reader (BioTek, Winooski, VT, USA).

### Metabolic cage

The O_2_ consumption, RER, and heat production were detected by Columbus Oxymax/CLAMS system (Columbus Instrument, Columbus, OH, USA). Experiments involved BAMBI Flox and BAMBI AKO mice with CD or HFD for 14 weeks. Mice were individually monitored for 48 h, and data were collected in a time interval of 30 min after 1-day adaption.

### Statistical analysis

All the replicate experiments (including cell and mouse-based experiments) are biological replicates that were repeated at least three times. All analyses involved the use of SPSS v23 (SPSS Inc, Chicago, IL). All data are represented as the mean ± SD. Comparison of two groups was determined by Student’s *t*-test and multiple groups by one-way ANOVA with Tukey’s post hoc test. The assumption of normality was tested by the Shapiro−Wilks test. All statistical tests were two-tailed, and *p* < 0.05 was considered statistically significant.

## Data availability

The data that support the findings of this study are available from the corresponding author upon reasonable request.

## Conflict of interest

The authors declare that they have no conflicts of interest with the contents of this article.
