# Supplementary material for: Adipose-specific BMP and activin membrane-bound inhibitor (BAMBI) deletion promotes adipogenesis by accelerating ROS production
Source: J Biol Chem. 2020 Nov 23;296:100037. doi: 10.1074/jbc.RA120.014793 (PMC7949090; doi:10.1074/jbc.RA120.014793)
Supplement: Figure S1 and Table S1 [file mmc1.docx]

**Figure S1**

**
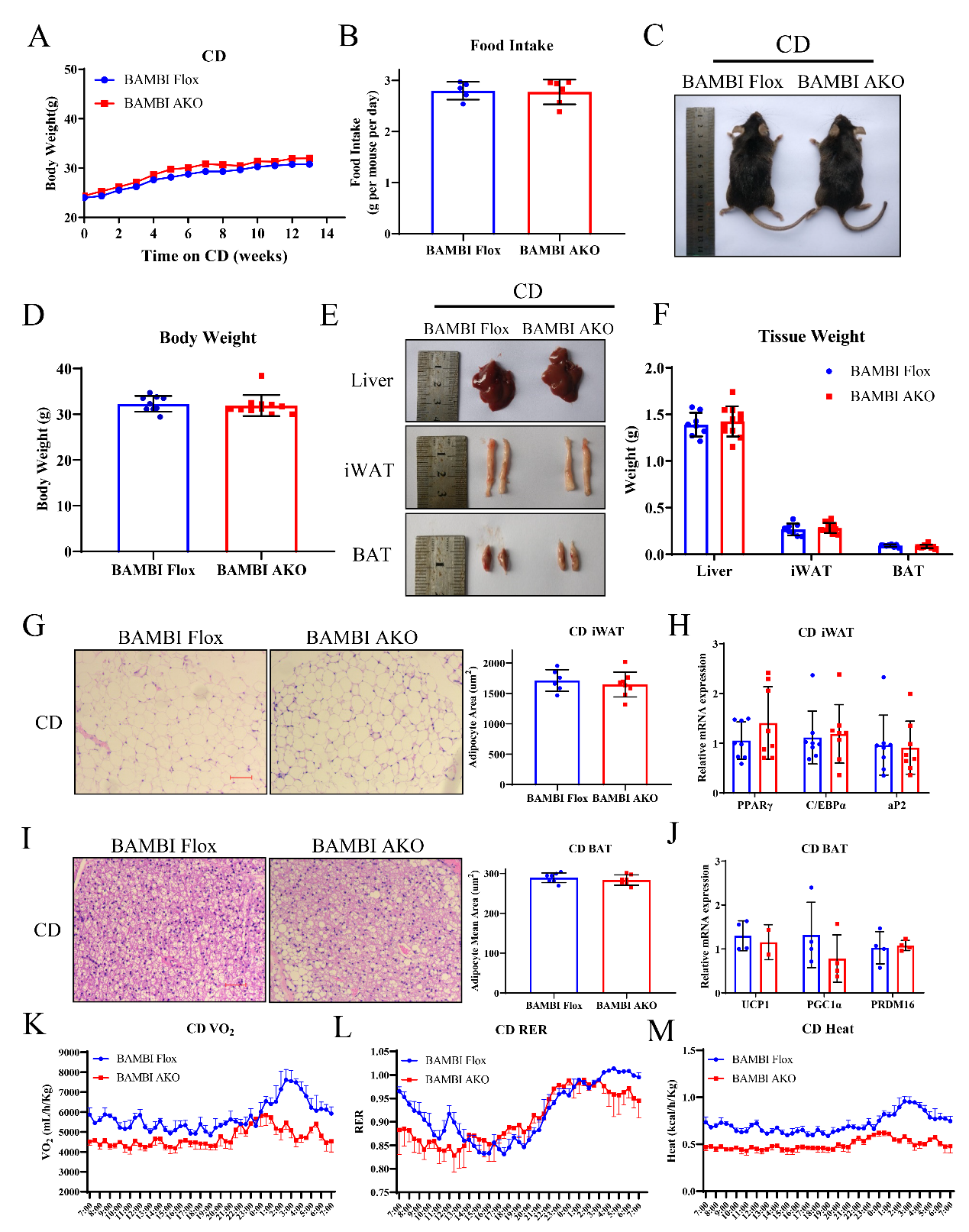
**

**Figure S1 BAMBI has no significant effect on adipogenesis with chow diet treatment.**

(A) Body weight and (B) food intake statistics of BAMBI Flox and AKO mice fed with chow diet (CD) for 14 weeks (n=12). (C) Comparison of body shape and (D) weight of mice after 14 weeks of CD feeding (n=10). (E) Morphological comparison and (F) weight statistics of liver, iWAT, eWAT and BAT after sacrifice of mice (n=10). (G) Representative image of H&E-stained sections and adipocyte area statistics of iWAT tissues from BAMBI Flox and BAMBI AKO mice fed with HFD diet. Scale bar: 50 μm. (H) Comparison of mRNA expression levels of PPARγ, C/EBPα, aP2 in iWAT of BAMBI Flox and AKO mice, with β-actin as a control (n=7). (I) Representative image of H&E-stained sections and adipocyte area statistics of BAT tissues from BAMBI Flox and AKO mice fed with HFD diet. Scale bar: 50 μm. (J) Comparison of mRNA expression levels of UCP1, PRDM16, and PGC1α in BAT of BAMBI Flox and AKO mice, with β-actin as a control (n=6). (K-M) Metabolism studies of control and BAMBI AKO mice fed an HFD: oxygen consumption (K), respiratory exchange ratio (RER) (L) and heat production (M) (n=4). Data are represented as the mean ± SD. Signiﬁcance was determined by t test analysis, *p < 0.05, **p < 0.001.

Table S1. The primer list of RT-qPCR

| Gene | Primer Sequences | |
| --- | --- | --- |
|  | Forward Primer | Reverse Primer |
| β-actin | GCCATGTACGTAGCCATCCA | ACGCTCGGTCAGGATCTTCA |
| PPARγ | CCAAGAATACCAAAGTGCGATCA | CCCACAGACTCGGCACTCAAT |
| aP2 | AAGAAGTGGGAGTGGGCTTTG | CTCTTCACCTTCCTGTCGTCTG |
| UCP1 | AGGCTTCCAGTACCATTAGGT | CTGAGTGAGGCAAAGCTGATTT |
| PRDM16 | CCACAAGTCCTACACGCAGT | GAGGGAGGAGGTAGTGCTGA |
| PGC1α | TATGGAGTGACATAGAGTGTGCT | CCACTTCAATCCACCCAGAAAG |
| ATGL | TTCGCAATCTCTACCGCCTC | AAAGGGTTGGGTTGGTTCAG |
| HSL | GCTGGGCTGTCAAGCACTGT | GTAACTGGGTAGGCTGCCAT |
| TNFα | TGTGCTCAGAGCTTTCAAAAC | GCCCATTTGAGTCCTTGATG |
| MCP-1 | CACTCACCTGCTGCTACTCAT | ATTCCTTCTTGGGGTCAGCAC |
| CD68 | GGGGCTCTTGGGAACTACAC | GTACCGTCACAACCTCCCTG |
| ADGRE1 | AACATGCAACCTGCCACAAC | TTCACAGGATTCGTCCAGGC |
| Bcl2 | ACGTGGACCTCATGGAGTG | TGTGTATAGCAATCCCAGGCA |
| Bax | TGAAGACAGGGGCCTTTTTG | AATTCGCCGGAGACACTCG |
| Nox4 | TGGCCAACGAAGGGGTTAAA | CCTAGGCCCAACATTTGGTGA |
| GCLC | CATGTTGGTGTCCTTCGATCATG | TGGTTGGGGTTTGTCCTTC |
| CyclinB | AAGGTGCCTGTGTGTGAACC | GTCAGCCCCATCATCTGCG |
| CyclinD | GCGTACCCTGACACCAATCTC | CTCCTCTTCGCACTTCTGCTC |
| CyclinE | GTGGCTCCGACCTTTCAGTC | CACAGTCTTGTCAATCTTGGCA |
| p21 | CCTGGTGATGTCCGACCTG | CCATGAGCGCATCGCAATC |
| IL-6 | TAGTCCTTCCTACCCCAATTTCC | TTGGTCCTTAGCCACTCCTTC |
| IFNγ | AGACAATCAGGCCATCAGCA | TGGACCTGTGGGTTGTTGAC |
